# Interaction of energy and sulfur microbial diet and smoking status with polygenic variants associated with lipoprotein metabolism

**DOI:** 10.3389/fnut.2023.1244185

**Published:** 2023-10-04

**Authors:** Haeng Jeon Hur, Hye Jeong Yang, Min Jung Kim, Kyunhee Lee, Dai Ja Jang, Myung-Sunny Kim, Sunmin Park

**Affiliations:** ^1^Food Functionality Research Division, Korea Food Research Institute, Wanju-gun, Jeollabuk-do, Republic of Korea; ^2^Department of Food Biotechnology, University of Science & Technology, Wanju-gun, Jeollabuk-do, Republic of Korea; ^3^Department of Food and Nutrition, Obesity, Diabetes Research Center, Hoseo University, Asan-si, Republic of Korea; ^4^R&D, Yejunbio, Asan-si, Republic of Korea

**Keywords:** hypo-HDL-cholesterolemia, cardiovascular diseases, energy intake, sulfur microbial diet, triglycerides, reverse cholesterol transport

## Abstract

**Introduction:**

Hypo-high-density lipoprotein cholesterolemia (hypo-HDL-C) contributes to the development of cardiovascular diseases. The hypothesis that the polygenic variants associated with hypo-HDL-C interact with lifestyle factors was examined in 58,701 middle-aged Korean adults who participated in the Korean Genome and Epidemiology Study (KoGES).

**Methods:**

Participants were categorized into the Low-HDL (case; *n* = 16,980) and Normal-HDL (*n* = 41,721) groups. The participants in the Low-HDL group were selected using the guideline-based cutoffs for hypo-HDL-C (<40 mg/dL for men and < 50 mg/dL for women) and included those taking medication for dyslipidemia. The genes associated with hypo-HDL-C were determined through a genome-wide association study (GWAS) in a city hospital-based cohort, and the results were validated in the Ansan/Anung study. The genetic variants for the single nucleotide polymorphism (SNP)-SNP interaction were selected using a generalized multifactor dimensionality reduction analysis, and the polygenic risk score (PRS) generated was evaluated for interaction with lifestyle parameters.

**Results:**

The participants with hypo-HDL-C showed a 1.45 and 1.36-fold higher association with myocardial infarction and stroke, respectively. The High-PRS with four SNPs, namely *ZPR1*_rs3741297, *CETP*_rs708272, *BUD13*_rs180327, and *ALDH1A2*_rs588136, and that with the 11q23.3 haplotype were positively associated with hypo-HDL-C by about 3 times, which was a 2.4-fold higher association than the PRS of 24 SNP with *p* < 5×10^−8^. The risk alleles of *CETP*_rs708272 and *ALDH1A2*_rs588136 were linked to increased expression in the heart and decreased in the brain, respectively. The selected SNPs were linked to the reverse cholesterol transport pathway, triglyceride-rich lipoprotein particle remodeling pathway, cholesterol storage, and macrophage-derived foam cell differentiation regulation. The PRS of the 4-SNP model interacted with energy intake and smoking status, while that of the haplotype interacted with a glycemic index of the diet, sulfur microbial diet, and smoking status.

**Discussion:**

Adults with a genetic risk for hypo-HDL-C need to modulate their diet and smoking status to reduce their risk.

## Introduction

Dyslipidemia, defined as an imbalance of lipids including cholesterol, low-density lipoprotein cholesterol (LDL-C), triglycerides (TG), and high-density lipoprotein cholesterol (HDL-C), is a risk factor for cardiovascular disease (CVD) worldwide (1). The global burden of dyslipidemias has increased over the last 30 years (2). In Korea, the incidence has risen from 9.0% in 2007 to 20.7% in 2018 (3). Dyslipidemia is highly prevalent in patients with metabolic syndrome (MetS) and type 2 diabetes (T2D), and this combination further increases the CVD risk (2). Hypo-HDL-cholesterolemia (hypo-HDL-C), hyper-LDL-cholesterolemia (hyper-LDL-C), and hypertriglyceridemia are influenced by a varied set of genetic and environmental factors (4). Therefore, the genetic factors and their interaction with lifestyle factors that influence hypo-HDL-C development may differ among Asians.

Cholesterol and triglycerides are emulsified with proteins and phospholipids to form lipoproteins that serve as vehicles for transporting cholesterol throughout the body. Under normal circumstances, LDL-C transports cholesterol into the peripheral tissues. However, when LDL-C is in excess, it can deposit cholesterol into the blood vessels, especially arteries, leading to a series of inflammatory changes in the vessel wall, resulting in CVD. HDL-C is synthesized in the liver and small intestines as ‘nascent’ HDL and contains a variety of lipid binding proteins called apolipoprotein (Apo) and includes ApoA1, ApoA2, ApoA4, ApoA5, ApoC1, ApoC2, ApoC3, and ApoE. ‘Nascent’ HDL is secreted into the bloodstream via transporter ATP Binding Cassette Subfamily A Member 1 (ABCA1) (5). The Apo proteins play specific roles in the reverse cholesterol transport function of HDL (5). ApoA1 and ApoC1 activate the enzyme lecithin–cholesterol acyl transferase (LCAT), and ApoA5, ApoC2, and ApoC3 modulate lipoprotein lipase (LPL) activity. The liver can also uptake HDL-C through ApoE, although it is a well-known ligand for the LDL receptor. The ‘mature’ HDL-C mediates cholesterol transport from non-hepatic tissues, especially arteries, to the liver. The returned cholesterol is metabolized and excreted through the bile (5). Therefore, reverse cholesterol transport via apolipoproteins in HDL-C is essential to reduce plaque formation in the blood vessels.

Plaque formation occurs in the blood vessels with the accumulation of LDL-C and its subsequent oxidation, followed by the inflammatory process of recruitment of monocytes-macrophages, uptake of oxidized LDL-C, and transformation of macrophages into foam cells. On the other hand, HDL-C particles protect against plaque formation by removing lipid buildup from the vessels and preventing inflammation (6). Hypo-HDL-C in persons without a history of CVD is inversely associated with future CVD risk, especially atherosclerosis. However, the inverse association may not apply to all patients with metabolic disorders and a history of CVD (7). Furthermore, serum HDL-C concentration may not always accurately represent the benefits of HDL function. This suggests that the functionality of HDL in reverse cholesterol transport can be influenced differently by various HDL subclasses with distinct particle sizes and compositions rather than solely by serum HDL concentration (8). However, the guideline for CVD includes that serum HDL-C should be maintained at a high level through lifestyle modifications.

Since genetic backgrounds significantly influence serum HDL-C, the interaction of genetic variants with lifestyle factors should be considered to modulate HDL-C. Several genetic variants have been reported to be associated with hypo-HDLC. These include variants associated with the following genes: low-density lipoprotein receptor (*LDLR*), proprotein convertase subtilisin-kexin type 7 (*PCSK7*), *APOA5*, SID 1 transmembrane family member 2 (*SIDT2*), and *ABCA1*, cholesterol ester transfer protein (*CETP*), *APOA1,* tyrosine-protein phosphatase non-receptor type 11 (*PTPN11*), rabphilin 3A *(RPH3A)*, and oligoadenylate synthetase 3 (*OAS3*) (9–11). However, studies on the role of single genetic variants that affect hypo-HDL-C have been conducted only with small sample sizes. A few studies have attempted to explore genetic variants and their interactions to evaluate the role of the polygenic risk score (PRS) in HDL-C function and the PRS interaction with lifestyle factors to influence hypo-HDL-C. We hypothesized that the polygenic variants associated with hypo-HDL-C interacted with lifestyle factors. The hypothesis was examined in 58,701 middle-aged Korean adults who participated in the Korean Genome and Epidemiology Study (KoGES) and validated in 13,598 adults in the combined regional and rural cohorts. The results can be used to modulate lifestyle factors to prevent hypo-HDL-C in genetically susceptible adults at risk of CVD.

## Methods

### Participants and setting

The KoGES aimed to establish a scientific basis for the implementation of customized treatments and preventive medicine by identifying risk factors for chronic diseases common among Koreans. Among several cohorts in KoGES, a large city hospital-based cohort (*n* = 58,701) and the Ansan/Ansung plus rural cohorts (*n* = 13,598) included the measurement of genetic variants, and their volunteers were used as the participants in the present study. The Ansan/Ansung plus rural cohorts were used as a replicate study for the genetic result. The participants aged 40–74 years were recruited during the years 2010–2014 (12). The institutional review boards (IRB) of the Korea National Institute of Health and Hoseo University approved the KoGES and the present study (KBP-2015-055 and 1041231-150811-HR-034-01, respectively). All participants signed written informed consent.

### Demographic, anthropometric, and biochemical parameters of the participants

On their initial visit to the hospital, the participants filled out survey forms for demographic information and lifestyles. Gender, education (<, =, or > high school), income (<monthly 2,000, 3,000, 4,000, or over 5,000 USD), physical activity, and smoking history were collected as the categorical variables. Alcohol and coffee consumption was recorded during a health interview. Current and past smokers were defined as smoking at least 20 cigarettes in the past six months and not smoking for at least the past six months, respectively (13). Daily alcohol and coffee intakes were calculated by multiplying the frequency of consumption by the amount consumed at one time (13). Regular physical activity was defined as more than 30 min of moderate physical activity for three or more days per week.

Height and weight were measured as described previously (12). Body fat and skeletal muscle masses were estimated using a prediction model generated by a machine learning algorithm from the Ansan/Ansung cohort based on the measurements made using the Inbody 3.0 (Cheonan, Korea) equipment, which uses the bioelectric impedance analysis method (14). The skeletal muscle index (SMI) was calculated by dividing the appendicular skeletal muscle mass (ASM) by height squared. Insulin resistance was calculated with homeostatic model assessment for insulin resistance (HOMA-IR), and it was also predicted using a prediction model made from a machine learning approach (15). The average systolic blood pressure (SBP) and diastolic blood pressure (DBP) were measured three times with a sphygmomanometer under resting conditions. After fasting for more than 12 h, the serum total cholesterol, HDL-C, triglycerides, creatinine concentrations, alanine aminotransferase (ALT) and aspartate aminotransferase (AST) activities, and plasma glucose concentrations were measured using a Hitachi 7,600 Automatic Analyzer (Hitachi, Tokyo, Japan). Blood HbA1c and serum high-sensitive C-reactive protein (hs-CRP) were measured using a ZEUS 9.9 automatic analyzer (Takeda, Tokyo, Japan) and a high-sensitivity ELISA kit (Thermofisher, Waltham, MA, USA), respectively.

### Definition of hypo-HDL-C

Hypo-HDL-C was defined as HDL-C < 40 mg/dL for men (*n* = 4,173) and < 50 mg/dL for women or the current use of anti-dyslipidemic medication (*n* = 12,807) (16). Participants were categorized into the Low-HDL and Normal-HDL groups based on the above definition. There were 16,980 and 41,721 participants in the Low-HDL and Normal-HDL groups, respectively.

### Food intake using a semi-quantitative food frequency questionnaire (SQFFQ)

The usual food intake during the past 12 months was measured using an SQFFQ with 106 food items commonly consumed by Koreans and validated with three-day food records of the four seasons (16, 17). Food intake was calculated by multiplying the frequency of each food consumption item by the amount consumed daily, as described previously. The food intake was expressed as grams/day. The daily intake of energy, carbohydrates, fats, proteins, vitamins, and minerals was calculated from the SQFFQ results using the computer-aided nutritional analysis program CAN-Pro 2.0 designed by the Korean Nutrition Society.

### Dietary patterns by principal component analysis, dietary inflammatory index (DII), glycemic index (GI), and sulfur microbial diet index

As reported previously, food items in the SQFFQ were divided into 30 predefined food groups, which were used to constitute the dietary patterns using principal component analysis (PCA). Based on eigenvalues >1.5 and the orthogonal rotation procedure (varimax), four dietary patterns were defined (15). The name of each dietary pattern was assigned to foods with ≥0.40 factor-loading values or predominant contributors (15). Supplementary Table S1 lists the foods in each dietary pattern. The groups were named the Korean-balanced diet (KBD), plant-based diet (PBD), Western-style diet (WSD), or rice-based diet (RBD) groups.

DII is an index of the pro-inflammatory potential of dietary components. As the intake of garlic, ginger, saffron, and turmeric was not recorded, they were excluded from the DII computation. DII was calculated by multiplying the dietary inflammatory scores of the 38 food and nutrient components by their daily intakes, and the sum of 38 items was divided by 100, as described previously (18).

The GI and glycemic load (GL) were calculated using the relevant equations. The GI of the same food can vary due to differences in the types of the food and its nutritional composition. The GI values listed for common Korean foods were used (19). The 43 gut microbes related to sulfur metabolism were selected, and food groups positively or negatively associated with the gut microbes were identified in a previous study (20). The food groups with a positive association were processed meats, liquor, and low-calorie drinks, and those with a negative association were beer, fruit juices, legumes, other vegetables, and sweets or desserts. Sulfur microbial diet scores were calculated by summing the multiplying value of the beta coefficient by the amount of each food item (20).

### Genotyping using a Korean Chip, quality control, and genome-wide association study (GWAS)

The participants’ genotypes in the Ansan/Ansung, rural, and city hospital-based cohorts were measured at the Center for Genome Science at the Korea National Institute of Health. The genotypes were measured in the genomic DNA isolated from whole blood using a Korean Chip (Affymetrix, Santa Clara, CA) designed for assessing the disease-related single nucleotide polymorphisms (SNPs) in Koreans (18). The inclusion criteria were ≥ 98% genotyping accuracy, <4% missing genotype call rate, <30% heterozygosity, and no gender bias. The genetic variants met the Hardy–Weinberg equilibrium (HWE) criterion at *p* > 0.05 and minor allele frequency (MAF) at >1% (18).

GWAS was conducted between the Low-HDL (*n* = 16,980) and Normal-HDL (*n* = 41,721) groups using the PLINK open-source whole genome association analysis toolset. The Manhattan and quantile-quantile (Q-Q) plots showed the quality of the selected genetic variants from the GWAS using the Fastman library in the R program (12). The Manhattan plot displayed the negative logarithms of the association *p*-values for each serum HDL-C concentration. The Q-Q plot displayed the quantile distribution of observed *p*-values (on the y-axis) versus the expected *p*-values (on the x-axis) for the genetic variants between the Low-HDL and Normal-HDL groups. The Q-Q plot indicated the goodness of fit between the actual and theoretical data distributions, and the lambda value of the Q-Q plot was calculated. The pathways linked to the genetic variants for serum HDL-C concentrations were selected using the MAGMA gene-set analysis in the SNP2GENE of the FUMA web application, available through the git repository.^1^ The statistical analysis was selected at *p*-values for the Bonferroni correction <0.05.

### Selection of the genetic variants to influence hypo-HDL-C and the optimal model with SNP-SNP interactions

**Figure 1:**
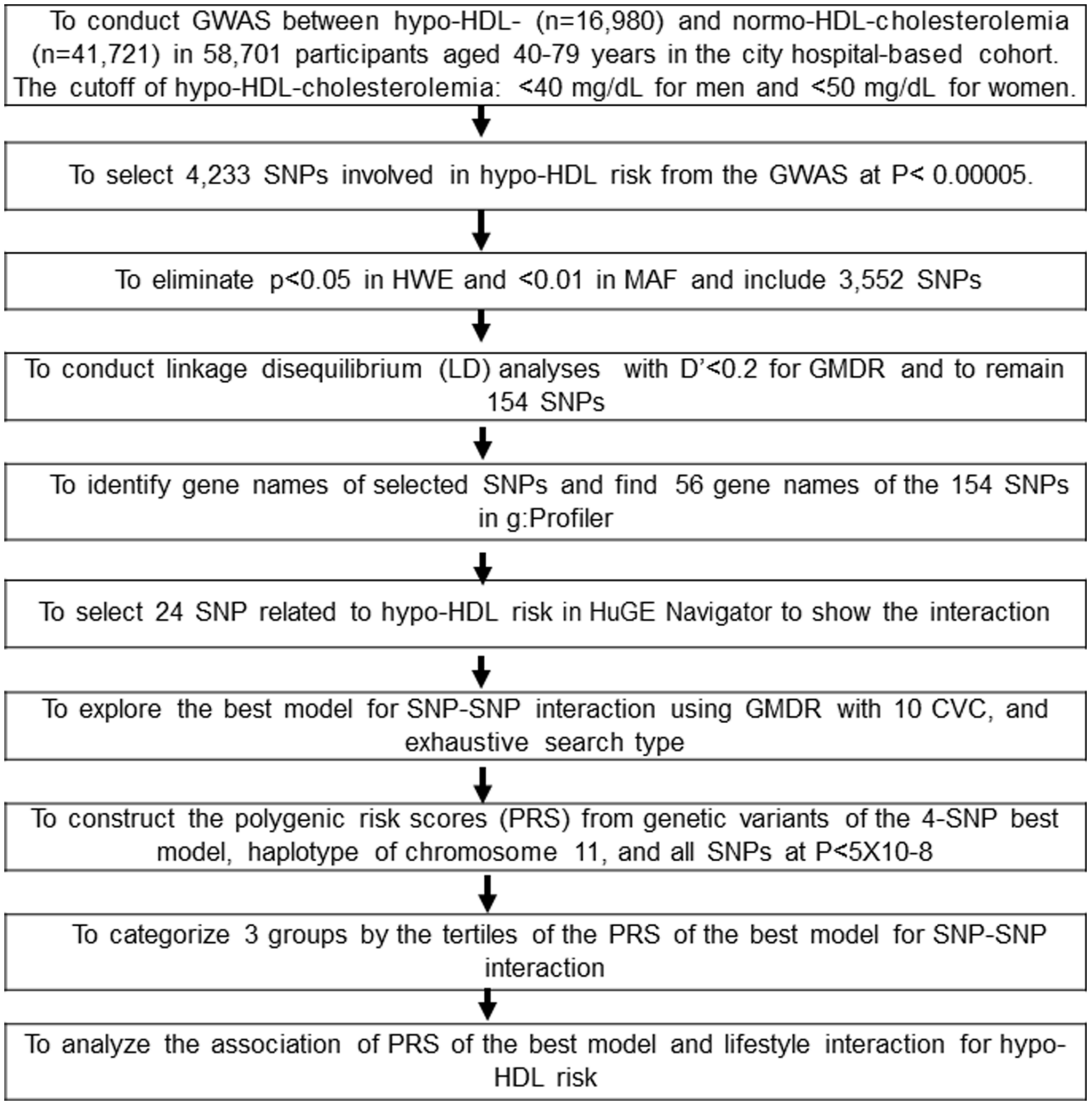
Flow chart to generate the polygenic risk score (PRS) associated with hypo-HDL-cholesterolemia by SNP-SNP interaction and haplotype and its interaction with lifestyle factors. Korean adults aged over 40 were categorized based on the guidelines based on the cutoff of HDL-C < 40 mg/dL for men and < 50 mg/dL for women, plus the current use of anti-dyslipidemic medication in the period 2010–2014. There were 16,980 and 41,721 participants in the Low-HDL and Normal-HDL groups, respectively.

The procedure to select genetic variants for hypo-HDL-C risk and to generate the best model with the SNP-SNP interactions is presented in Figure 1. Genetic variants associated with hypo-HDL-C risk were evaluated to select 4,233 SNPs in the urban hospital-based cohort (*p* < 5×10^−5^). Among the genetic variants, those not meeting the criteria for HWE and MAF were removed (*n* = 681). The linkage disequilibrium (LD) analyses were conducted on the SNPs of the 3,552 genetic variants in the same chromosome using Haploview 4.2 in PLINK. The genetic variants having an LD score of D′ ≥ 0.2 were eliminated because they provided the same information on the genetic impact. The gene names of the remaining 154 genetic variants were searched using g:Profiler,^2^ and 56 SNPs were identified by gene names. The pathways involved in the genetic variants were identified, and 24 SNPs were selected.^3^ The optimal SNP-SNP interaction model was identified using the generalized multifactor dimensionality reduction (GMDR).

Ten genetic variants interacted with each other and were selected by GMDR from among the 24 genetic variants associated with hypo-HDL-C risk. The optimal SNP-SNP interaction model was selected in a sign rank test of trained balanced accuracy (TRBA) and testing balanced accuracy (TEBA) while adjusting for the covariates using a GMDR program and a *p*-value threshold of 0.05 (12). The covariates used were age, gender, residence area, body mass index, education, and income for model 1, and the model 1 covariates plus energy intake, alcohol intake, regular exercise, and smoking status for model 2. The ten-fold cross-validation was also checked for cross-validation consistency (CVC) because the sample size was larger than 1,000 (12). The 10 out of 10 scores in the CVC indicated perfect cross-validation criteria.

### Haplotype analysis and polygenic risk score

The haplotype was considered to show the genetic impact of the hypo-HDL-C risk when the primary genetic variants were located on the same chromosome. The LD of the selected SNP met the criteria (D′ < 0.2). The haplotypes and their frequencies were analyzed using the GPLINK software (21).

The risk allele number of each SNP was counted to generate the PRS of the optimal models. For example, the genetic score for the SNP was 2, 1, and 0 when the participants had AA, AG, and GG of one SNP, respectively, and the A allele was the risk allele. The PRS of the best model was assessed by summing the number of the risk alleles from each selected SNP in the best gene–gene interaction model (21, 22). The PRSs in the three and six SNP models were divided into three categories according to the number of risk alleles. They were classified as Low-PRS, Middle-PRS, and High-PRS when the number of risk alleles in the PRS was 0–2 (*n* = 19,686), 3–4 (*n* = 30,513), and ≥ 5 (*n* = 3,629) in the three-SNP model and 0–5 (*n* = 27,212), 6–7 (*n* = 20,375), and ≥ 8 (*n* = 1,822) in the six-SNP model, respectively. Among the best models to meet the value of *p* of the sign test and CVC, the model with the lowest SNP number (three-SNP model) was used to explore its interaction with the lifestyle parameters.

### Expression quantitative trait locus (eQTL) analysis

The eQTL analysis is a direct approach to estimating the candidate gene expression with the genetic variants at risk loci. Gene expressions corresponding to the genetic variants related to the hypo-HDL risk were determined by eQTL analysis in the Genotype-Tissue Expression (GTE) × eQTL calculator.^4^

### Molecular docking of the gene having missense mutation with food compounds and molecular dynamics simulation (MDS)

The wild and mutated protein structures were generated in the *Protein Data Bank* (PDB) format from the Iterative Threading Assembly Refinement (I-TASSER) website.^5^ The proteins were switched into the PDB, partial charge (Q), and atom type (T) (PDBQT) files using AutoDock Tools 1.5.6 (Molecular Graphics Laboratory, Scripps Research Institute, FL, USA) (23). The active sites of the proteins were searched using the ProteinsPlus website.^6^ The active functional pockets and the mutated sites were also included in the active site for molecular docking. Food compounds (*n* = 20,000) were converted to the PDBQT file format, and water molecules attached to the ligands were removed (23). Food components having < −10 kcal/mol binding energy between the proteins and food components were selected (24). The lower the binding free energy, the tighter the binding and affinity.

The conformational changes in the protein structures were examined using MDS to detect the changes in their activity. After the top docking poses with the selected food components were added, simulations were conducted on the docked complexes between the protein and food components. The Chemistry at Harvard Macromolecular Mechanics (CHARMM) force field was added to the docked complex in the “Simulation” part, and the protein was solvated by “Solvation.” The “Standard Dynamics Cascade” was used to set the molecular dynamics simulation parameters for the protein added to the solvent system. The root mean square deviation (RMSD), root mean square fluctuations (RMSF), and hydrogen bond values were determined after the 10 ns simulation.

### Statistical analysis

Statistical analysis was performed using SAS (version 9.3; SAS Institute, Cary, NC, USA). The sample size was determined by satisfying the significance at *α* = 0.05, *β* = 0.99, and 1.05 odds ratio in the logistic analysis using a G-power calculator. The sample size of 57,801 was sufficient to achieve the significance. Frequency distributions were used for the descriptive statistics for categorical variables between the Low-HDL and Normal-HDL groups, and a Chi-square test was applied to determine statistical significance. Descriptive statistics of the continuous variables were determined as the adjusted means with standard deviations after adjusting for the covariates linked to dyslipidemia. The gender and HDL groups were used as the main effects, and their interactions were evaluated in a two-way analysis of covariance (ANCOVA) (19). Multiple comparisons of the groups were conducted using Tukey’s test.

The association of hypo-HDL-C with the biochemical parameters was evaluated using a logistic regression analysis after adjustment for covariates. The odds ratios (ORs) and 95% confidence intervals (CIs) of hypo-HDL-C with each biochemical parameter were calculated. The covariate set 1 was age, residence area, survey year, body mass index (BMI), education, and income. Set 2 was the covariates in set 1, plus energy intake, physical activity, smoking status, and alcohol consumption, and the covariate set 3 were covariates of set 2, plus blood HbA1c and serum triglyceride concentration. In the two-way analysis of covariance (ANCOVA), when the interaction terms between the PRS and lifestyle-related parameters were statistically significant, each lifestyle-related parameter was categorized into the Normal-HDL or Low-HDL groups with the designated cutoff. The adjusted odds ratio (ORs) and 95% confidence intervals (CIs) of hypo-HDL-C with PRS were also calculated by adjusted logistic regression analysis with covariate set 3 between the Normal-HDL and Low-HDL groups. The significant differences between the Low-HDL and Normal-HDL groups were analyzed using the χ^2^ test in the low- and high groups of lifestyle-related parameters.

## Results

### Characteristics of the participants

**Figure 2:**
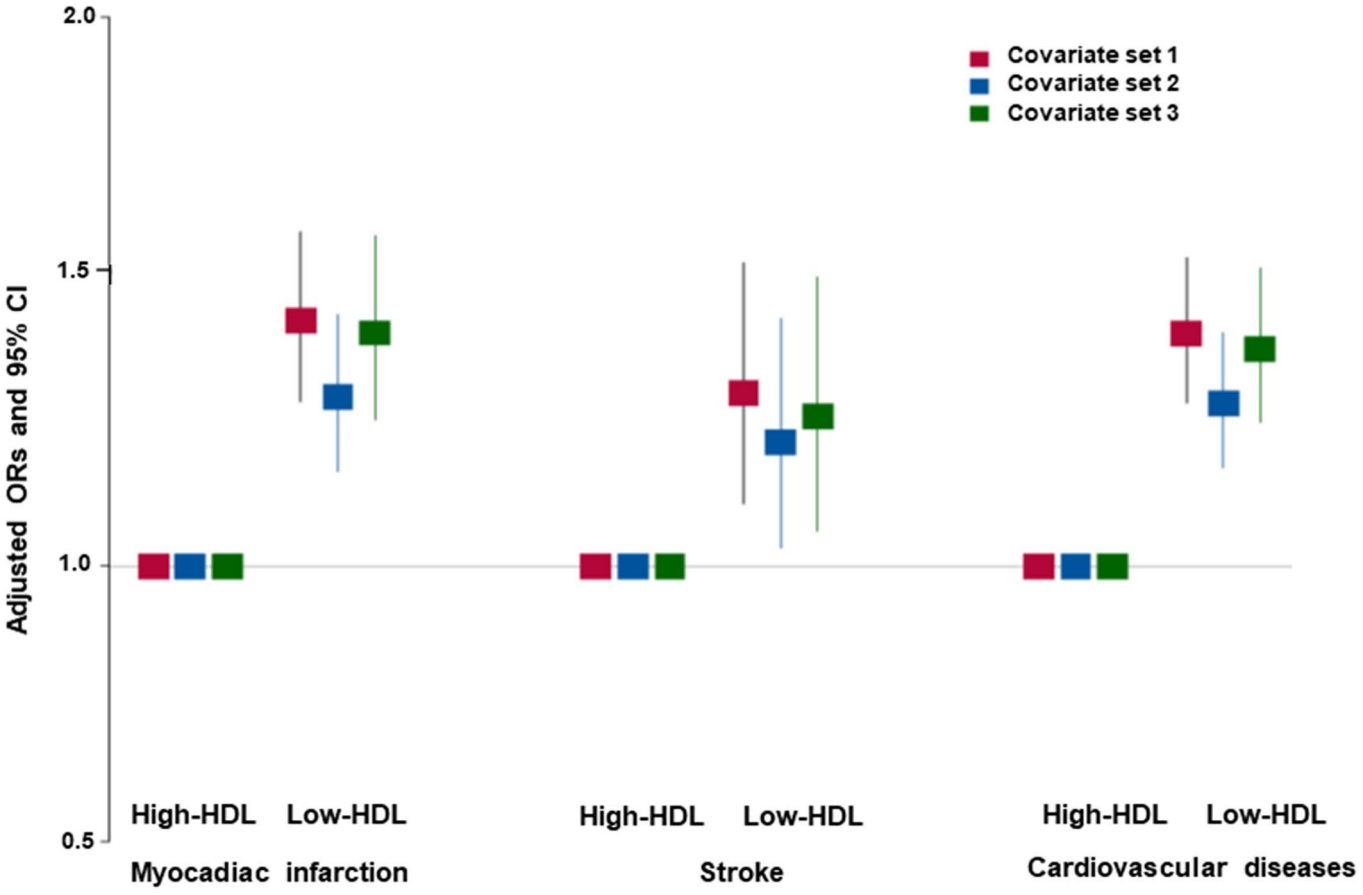
Adjusted odds ratio (ORs) and 95% confidence intervals (CIs) of hypo-HDL-cholesterolemia with cardiovascular diseases. Covariates set 1 included age, gender, body mass index, residence area, education, and income; covariate set 2 contained those in set 1 plus energy intake, exercise, alcohol consumption, smoking, and incidence of osteoporosis; and covariate set 3 included those in set 2 plus blood HbA1c and serum triglyceride concentrations.

The participants in the Low-HDL group were older, less educated, and earned a lower income than those in the Normal-HDL group, but this difference was restricted only to the women (Table 1). The participants in the Low-HDL group had higher BMI, waist circumferences, and fat mass than those in the Normal-HDL group for both genders, but only women had a lower SMI (Table 1). Serum glucose and blood HbA1c concentrations and insulin resistance were higher in the participants in the Low-HDL group than those in the Normal-HDL group. The participants in the Low-HDL group had a higher incidence of dyslipidemia and hypertension (Table 1). The incidence of myocardial infarction, stroke, and cardiovascular disease was higher in the Low-HDL group than in the Normal-HDL group for both genders (Table 1). The participants in the Low-HDL group were higher at 1.4 times the risk of cardiovascular diseases, including myocadiac infarction and cerebrovascular stroke, than the Normal-HDL (Figure 2).

**Table 1:** General, anthropometric, and biochemical characteristics according to gender and hypo-HDL.

	Men (*n* = 20,293)		Women (*n* = 38,408)	
	Normal-HDL (*n* = 16,120)	Low-HDL (*n* = 4,173)	Normal-HDL (*n* = 25,601)	Low-HDL (*n* = 12,807)
Age (years)	57.1 ± 0.09^a^	57.3 ± 0.17^a^	52.1 ± 0.07^c^	53 ± 0.1^b***+++^
Education (*N*, %)				
≤Middle school	1,356 (13.8)	397 (14.9)	3,868 (19.8)	2,784 (26.4)^‡‡‡^
High school	7,474 (76.0)	1960 (74.6)	14,388 (73.8)	7,234 (68.7)
≥Collage	1,015 (10.3)	276 (10.5)	1,248 (6.4)	518 (4.92)
Income (*N*, %)				
≤$2000	1,228 (8.11)	355 (9.06)	2,410 (10.1)	1705 (14.4) ^‡‡‡^
$2000–4,000	6,401 (42.3)	1,690 (43.1)	10,402 (43.6)	5,400 (45.5)
>$4,000	7,507 (49.6)	1874 (47.8)	11,073(46.4)	4,761(40.1)
BMI (kg/m^2^)	24.3 ± 0.04^b^	25.2 ± 0.06^a^	23.2 ± 0.03^c^	24.2 ± 0.04^b***+++^
Waist circumferences(cm)	84.8 ± 0.08^b^	87.6 ± 0.13^a^	77.3 ± 0.05^d^	80.2 ± 0.07^c***+++^
SMI (kg/cm)	7.17 ± 0.005^a^	7.19 ± 0.01^a^	6.12 ± 0.004^b^	6.07 ± 0.005^c***++###^
Fat mass (%) 25 32	22.5 ± 0.01^d^	22.7 ± 0.02^c^	31.4 ± 0.01^b^	31.6 ± 0.01^a***+++###^
Fasting glucose (mg/dL)	97.6 ± 0.25^b^	98.6 ± 0.46^a^	93.4 ± 0.2^d^	95.3 ± 0.27^c***+++^
HbA1c (%)	5.67 ± 0.01^c^	5.78 ± 0.02^b^	5.69 ± 0.01^c^	5.83 ± 0.01^a**+++^
Insulin resistance (*N*, %)	1,555 (9.79)	728 (17.7) ^‡‡‡^	982 (3.88)	1,289 (10.2) ^‡‡‡^
Total cholesterol (mg/dl)	192 ± 0.47^b^	176 ± 0.84^c^	206 ± 0.36^a^	192 ± 0.5^b***+++###^
HDL (mg/dl)	52.9 ± 0.09^c^	35.9 ± 0.16^d^	62.6 ± 0.07^a^	43.1 ± 0.09^b***+++^
LDL (mg/dl)	115 ± 0.43^c^	103 ± 0.78^d^	123 ± 0.33^a^	118 ± 0.46^b***+++###^
TG (mg/dl)	118 ± 1^c^	183 ± 1.8^a^	101 ± 0.77^d^	154 ± 1.08^b***+++##^
SBP (mmHg)	125 ± 0.14^a^	124 ± 0.24^b^	121 ± 0.1^c^	121 ± 0.14^c***++##^
DBP (mmHg)	78.2 ± 0.09^a^	77.3 ± 0.16^b^	74.5 ± 0.07^c^	74.4 ± 0.09^c***+++###^
Myocardial infarction (*N*, %)	636 (4.01)	228 (5.55)^‡‡‡^	420 (1.66)	370 (2.93) ^‡‡‡^
Stroke (*N*, %)	276 (1.74)	92 (2.24) ^‡^	170 (0.67)	144 (1.14) ^‡‡‡^
Cardiovascular disease (*N*, %)	890 (5.61)	315 (7.67) ^‡‡‡^	581 (2.30)	502 (3.97) ^‡‡‡^

Values represent adjusted means and standard errors for continuous variables and the number (*N*) and percentage for categorical variables. BMI, body mass index; SMI, skeletal muscle index (SMI defined as appendicular skeletal muscle mass/height); HbA1c, hemoglobin A1c; TG, triglyceride; SBP, systolic blood pressure; DBP, diastolic blood pressure. ^*^Significant differences by gender at *p* < 0.05, ^**^ at *p* < 0.01, ^***^ *p* < 0.001. ^#^Significant differences by height at *p* < 0.05, ^##^ at *p* < 0.01, ^###^ *p* < 0.001. ^+^Significant interaction between gender and height at *p* < 0.05, ^++^ at *p* < 0.01, ^+++^ *p* < 0.001. ^‡^Significantly different from the control group in *χ*^2^ test in each gender at *p* < 0.05, ^‡‡^ at *p* < 0.01, ^‡‡‡^ at *p* < 0.001. ^a,b,c,d^Different superscript letters indicated significant differences among the groups in Tukey’s test at *p* < 0.05.

### Lifestyles, including nutrient intake, and dietary patterns

There was no difference in the daily energy intake between the Low-HDL and the Normal-HDL groups. The participants of both genders in the Low-HDL group consumed higher carbohydrates and lower fat than those with Normal-HDL (Table 2). Protein, fiber, calcium, sodium, and vitamin D intakes were lower in the women participants with the Low-HDL group than in those with the Normal-HDL group (Table 2). The DII, GI of the food consumed, and flavonoid intake did not differ between the Low-HDL and Normal-HDL groups. Coffee intake was lower in the Low-HDL than the Normal-HDL group only in women, and alcohol intake was lower for both genders (Table 2). Fewer participants exercised regularly in the Low-HDL than in the Normal-HDL group for both genders, and the number of male smokers was much higher in the Low-HDL group (Table 2).

**Table 2:** Daily nutrient intake according to gender and hypo-HDL-cholesterolemia.

	Men (*n* = 20,293)		Women (*n* = 38,408)	
	Normal-HDL (*n* = 16,120)	Low-HDL (*n* = 4,173)	Normal-HDL (*n* = 25,601)	Low-HDL (*n* = 12,807)
Energy intake (EER %)	90.1 ± 0.40^b^	89.5 ± 0.73^b^	102 ± 0.31^a^	101 ± 0.44^a***^
CHO (En%)	71.8 ± 0.09^b^	72.6 ± 0.16^a^	71.1 ± 0.07^c^	72.2 ± 0.1^a***+++#^
Fat (En%)	13.7 ± 0.07^b^	13.1 ± 0.13^a^	14.4 ± 0.05^c^	13.4 ± 0.07^a***+++##^
SFA (En%)	8.45 ± 0.06^a^	7.90 ± 0.11^b^	8.02 ± 0.05^b^	7.39 ± 0.06^c^
MUFA (En%)	10.7 ± 0.08^a^	10.1 ± 0.13^b^	10.0 ± 0.05^b^	9.20 ± 0.07^c***+++^
PUFA(En%)	5.99 ± 0.05^a^	5.84 ± 0.08^a^	5.48 ± 0.03^b^	5.24 ± 0.04^c***+++^
Protein (%)	13.2 ± 0.03^c^	13.1 ± 0.06^c^	13.7 ± 0.03^a^	13.5 ± 0.04^b***+++^
Fiber (mg)	15.0 ± 0.1^a^	15.2 ± 0.16^a^	14.3 ± 0.07^b^	14.6 ± 0.09^a***+^
Ca (mg)	426 ± 3.5^c^	424 ± 6.31^c^	488 ± 2.7^a^	475 ± 3.77^b***+^
Na (mg)	2,512 ± 18.4^a^	2,593 ± 33.2^a^	2,372 ± 14.2^c^	2,437 ± 19.8^b***+++^
Vitamin C (mg)	99.5 ± 0.92^b^	102 ± 1.65^b^	115 ± 0.71^a^	116 ± 0.99^a***+^
Vitamin D (mg)	5.63 ± 0.06^c^	5.41 ± 0.10^c^	7.00 ± 0.04^a^	6.57 ± 0.05^b***+++^
DII	−20.1 ± 0.02^a^	−20.8 ± 0.04^a^	−21.1 ± 0.02^b^	−21.6 ± 0.02^b***+^
Flavonoids (ug)	34.1 ± 0.43^b^	35.2 ± 0.78^b^	43.7 ± 0.33^a^	43.4 ± 0.46^a***^
Glycemic index	51.0 ± 0.10^a^	51.1 ± 0.17^a^	47.6 ± 0.07^b^	47.5 ± 0.09^b***^
Sulfur microbial diet index	−34.6 ± 0.69^a^	−36.3 ± 1.18^a^	−55.2 ± 0.49^b^	−55.8 ± 0.66^b***^
Coffee (g/day)	3.72 ± 0.03^a^	3.71 ± 0.05^a^	3.73 ± 0.02^a^	3.39 ± 0.03^b***+++###^
Alcohol (g/week)	230 ± 4.67^b^	138 ± 8.5^a^	70.1 ± 3.63^c^	53.9 ± 5.07^d***+++###^
Exercise (*N*, %)	9,534 (60.2)	2,250 (54.8)^‡‡‡^	13,569(53.8)	6,257 (49.6)^‡‡‡^
Former smoking Smoking (*N*, %)	7,134 (44.4)	1,661 (39.9)^‡‡‡^	313 (1.23)	147 (1.15)
Smoking (*N*, %)	4,259 (21.0)	1,405 (33.7)	473 (1.24)	276 (2.16)

Values represent adjusted means and standard errors for continuous variables and the number and percentage for categorical variables. EER, estimated energy requirement; En%, energy percent; CHO, carbohydrates; SFA, saturated fatty acids; MUFA, monounsaturated fatty acid; PUFA, polyunsaturated fatty acid; DII, dietary inflammatory index; GI, dietary glycemic index. ^*^Significant differences by gender at *p* < 0.05, ^**^ at *p* < 0.01, ^***^ *p* < 0.001. ^#^ Significant differences by height at *p* < 0.05, ^##^ at *p* < 0.01, ^###^ *p* < 0.001. ^+^ Significant interaction between gender and height at *p* < 0.05, ^++^ at *p* < 0.01, ^+++^ *p* < 0.001. ^‡^ Significantly different from the control group in *χ*^2^ test in each gender at *p* < 0.05, ^‡‡^ at *p* < 0.01, ^‡‡‡^ at *p* < 0.001. ^a,b,c,d^ Different superscript letters indicated significant differences among the groups in Tukey’s test at *p* < 0.05.

### Genetic variants associated with hypo-HDL-C

The statistical significance of the genetic variants associated with hypo-HDL-C has been shown in a Manhattan plot (Supplementary Figure S1A). Lambda, a genome inflation factor for genetic variants linked to hypo-HDL-C, was calculated by comparing the observed and expected *p* values. This comparison was shown in the Q–Q plot, and the lambda was 1.083, indicating no inflation of the genetic variants (Supplementary Figure S1B).

**Figure 3:**
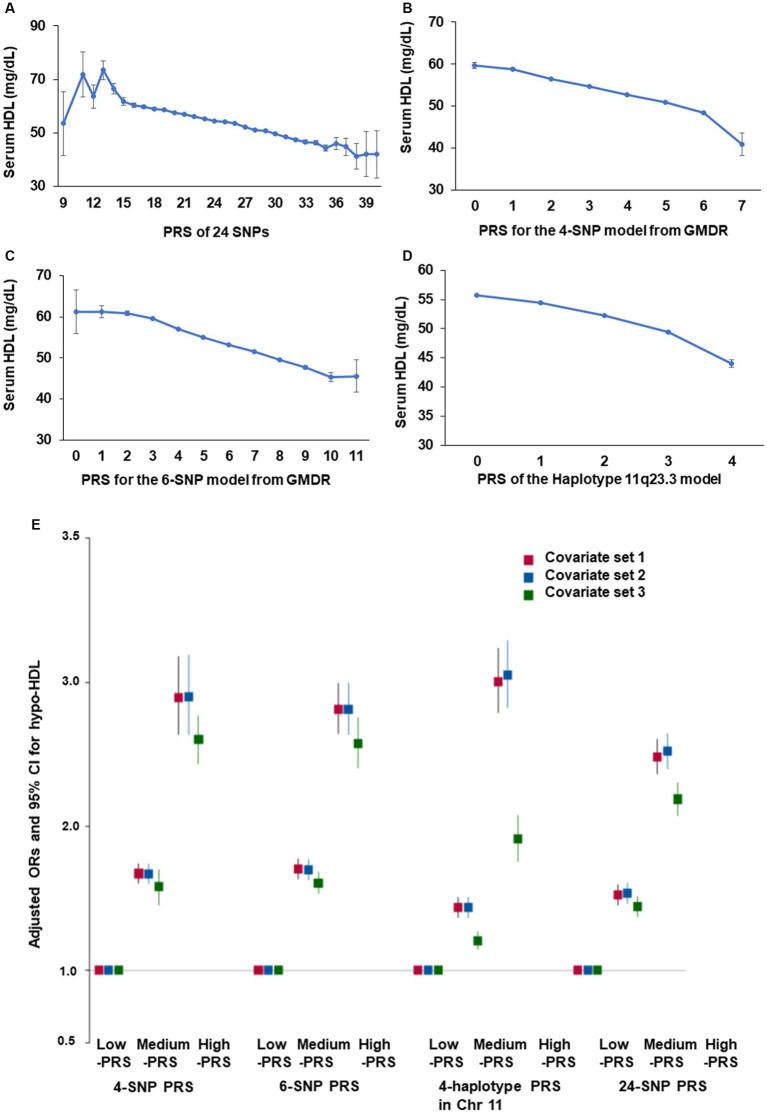
Adjusted odds ratio (ORs) and 95% confidence intervals (CIs) of hypo-HDL-C with polygenic risk score (PRS) generated by different methods. **(A)** Serum HDL-C concentration according to the PRS of all SNPs (24 SNPs) with *p* < 5×10^−8^. **(B)** Serum HDL-C concentration according to the PRS of 4 SNPs (*BUD13_*rs180327, *ZPR1*_rs3741297, *ALDH1A2*_rs588136, and *CETP*_rs708272) selected from the SNP-SNP interaction by generalized multifactor dimensionality reduction (GMDR). **(C)** Serum HDL-C concentration according to the PRS of 6 SNPs (4-SNP plus *LPL*_rs325 and *ABCA1*_rs1883025) selected from the SNP-SNP interaction by GMDR. **(D)** Serum HDL-C concentration according to the PRS of 4 SNPs in haplotype 11q23.3 selected from the SNP-SNP interaction by GMDR. **(E)** Adjusted odds ratio (ORs) and 95% confidence intervals (CIs) of hypo-HDL-cholesterolemia with PRS with 24 SNP, 4-SNP PRS. 6-SNP PRS, and PRS of the haplotype 11q23.3. PRS was generated as the sum of the number of risk alleles in each SNP generated from the SNP-SNP interaction and haplotype. They were classified as Low-PRS, Middle-PRS, and High-PRS according to the range 0–3, 4–5, and ≥ 6 in the four-SNP model and 0–5, 6–7, and ≥ 8 in the six-SNP model, respectively. Covariates set 1 included age, gender, body mass index, residence area, education, and income; covariate set 2 contained those in set 1 plus energy intake, exercise, alcohol consumption, smoking, and incidence of osteoporosis; and covariate set 3 included those in set 2 plus blood HbA1c and serum triglyceride concentrations.

The PRS of 24 genetic variants associated with hypo-HDL-C satisfied the inclusion criteria, such as *p* < 5×10^−8^ for the GWAS, D′ < 0.2 for LD, *p* ≥ 0.05 for HWE, and ≥ 0.01 for MAF. In the PRS containing the risk alleles of 24 genetic variants, serum HDL-C was lower in the high-PRS containing up to 12–15 risk alleles, but they continuously decreased with PRS containing up to 37 risk alleles (Figure 3A). Among the 24 genetic variants, ten were selected as having similar pathways and interactions with each other using the GeneMANIA site. The characteristics of the 10 genetic variants are shown in Table 3. These 10 genetic variants were associated with hypo-HDL-C at *p* < 5×10^−12^ in the city hospital-based cohort (*n* = 58,701) and at *p* < 5×10^−5^ in the Ansan/Ansung plus rural cohorts (*n* = 13,598).

**Table 3:** Characteristics of genetic variants related to adult height from generalized multifactor dimensionality reduction analysis.

CHR^1^	SNP^2^	Base pair	A1^3^	A2^4^	OR^5^	SE^6^	*p* for city^7^	*p* for asan+nong^8^	MAF^9^	*P* for HWE^10^	Gene names	Location
7	rs146148222	80,304,855	G	C	0.8192	0.02823	1.64E-12	3.35E-05	0.0702	0.8087	*CD36*	Intron
8	rs325	19,819,328	C	T	0.7257	0.02239	1.68E-46	2.41E-11	0.1249	0.3452	*LPL*	Intron
9	rs1883025	107,664,301	T	C	1.218	0.01593	3.26E-35	7.17E-07	0.2508	0.8261	*ABCA1*	Intron
11	rs180327	116,623,659	C	T	1.164	0.0141	3.7E-27	9.45E-09	0.4312	0.2257	*BUD13*	Intron
11	rs3741297	116,657,667	T	C	1.887	0.02421	1.1E-151	7.80E-16	0.0797	0.1767	*ZPR1*	Intron
11	rs5069	116,708,254	A	G	0.7417	0.03244	4.11E-13	4.42E-05	0.0541	0.936	*APOA1*	5’-UTR
11	rs7115583	11,678,437	T	G	0.8182	0.02029	4.61E-23	1.12E-06	0.1489	0.4839	*SIK3*	Intron
15	rs588136	58,730,498	C	T	0.819	0.01455	7.29E-43	1.33E-12	0.3930	0.869	*ALDH1A2*	Intron
16	rs708272	56,996,288	A	G	0.7472	0.01473	4.14E-87	6.74E-10	0.3816	0.1618	*CETP*	Intron
19	rs429358 (cys130arg)	45,411,941	C	T	1.309	0.02316	3.03E-31	2.74E-11	0.0956	0.5349	*APOE*	Missense

^1^Chromosome; ^2^Single nucleotide polymorphism; ^3^Minor allele; ^4^Major allele^5^Odds ratio (OR) for city cohort; ^6^Standard error; ^7^P-value for OR after adjusting for age, gender, residence area, survey year, body mass index, daily energy intake, education and income in the hospital-based cohort (case: *n* = 17,545; control: *n* = 36,283); ^8^P-value for OR after adjusting for covariates in the Ansan/Ansung cohort (case: *n* = 1,657; control: *n* = 3,245); ^9^Minor allele frequency; ^10^Hardy-Weinberg equilibrium.

### PRS for interacted genetic variants each other or the haplotype

The optimal model with genetic variants interacting with each other was found using GMDR. The 4-SNP and 6-SNP models met the criteria for TEBA at *p* < 0.05 and 10/10 CVC. The 4-SNP model included *BUD13_*rs180327, *ZPR1*_rs3741297, *ALDH1A2*_rs588136, and *CETP*_rs708272, and the 6-SNP model added *LPL*_rs325 and *ABCA1*_rs1883025 to the genetic variants of the 4-SNP model (Supplementary Table S2). Among the 10 genetic variants, 4 genetic variants in chromosome 11 were part of the haplotype, and its PRS was calculated. Four genetic variants in chromosome 11 showed D′ < 0.2 of LD, as shown in Supplementary Figure S2. As the PRS for the 4-SNP, 6-SNP, and haplotype 11q23.3 models decreased, the serum HDL-C was lowered (Figures 3B–D). However, the decline in serum HDL-C was greater in the 4-SNP model than in the 6-SNP model. In haplotype 11q23.3, the genetic variants with LD ≥ 0.2 were removed (Supplementary Figure S2).

After dividing the PRS for the 4-SNP and 6-SNP models, haplotype for chromosome 11, and 24 genetic variants, their high-PRS was associated with hypo-HDL-C compared to the Low-PRS by 2.899 (2.637–3.187), 2.81 (2.64–2.99), 3.048 (2.826–3.288), and 2.52 (2.402–2.644) times, in the 4-SNP, 6-SNP, haplotype, and 24-SNP models, respectively, after adjusting for covariates linked with dyslipidemia (Figure 3E). These results showed that the 4-SNP model and the haplotype were optimal for predicting genetic risk of hypo-HDL-C.

### Gene expression by eQTL according to genetic variants

**Figure 4:**
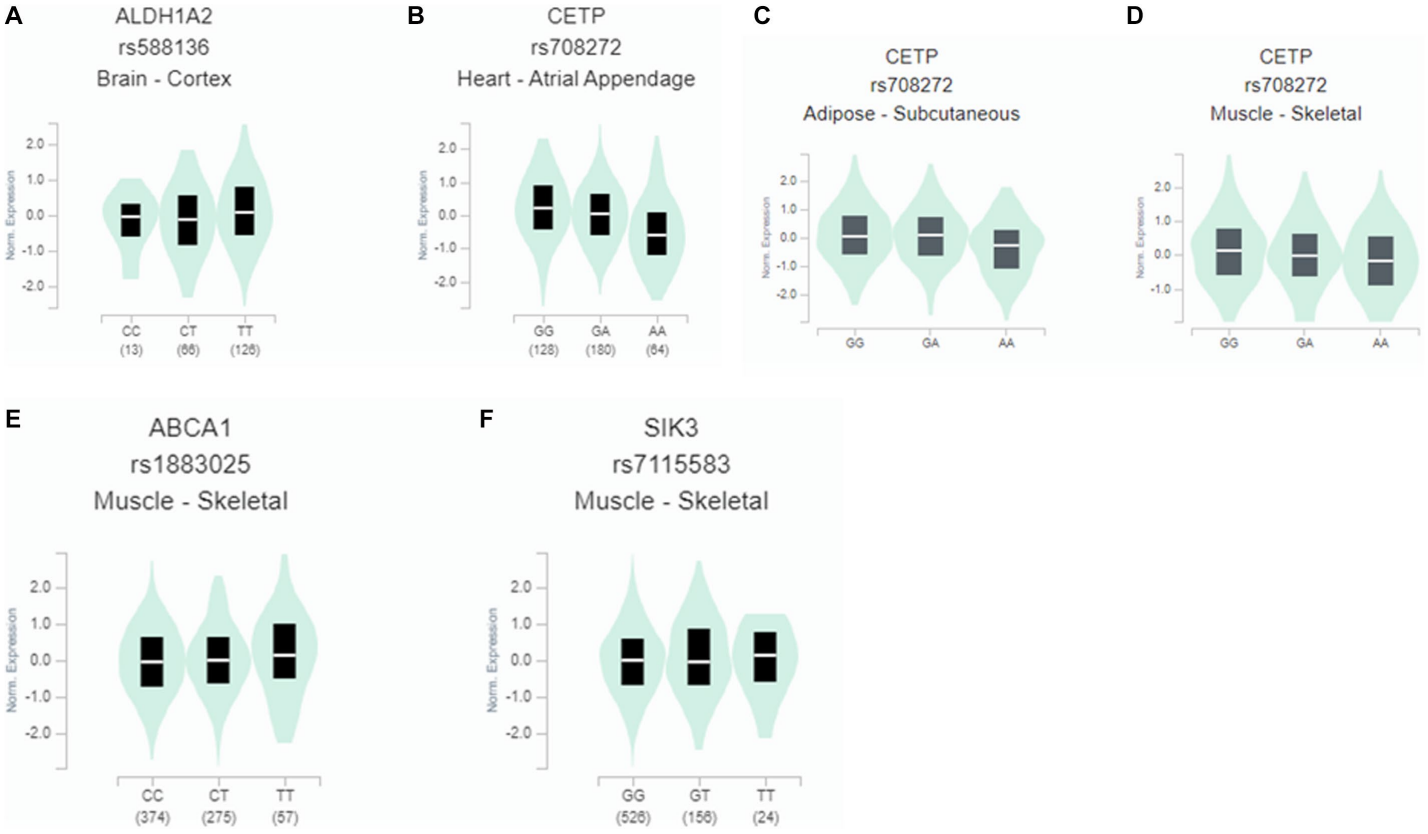
Gene expression according to the alleles of the selected SNPs for hypo-HDL-cholesterolemia risk in different tissues. **(A)** *ALDH1A2*_rs588136 in the cortex of the brain (*β* = 0.14; *p* = 0.03). **(B)** *CETP*_rs708272 in the arterial appendage (*β* = −0.31; *p* = 5.5×10-7). **(C)** *CETP*_rs708272 in the skeletal muscle (*β* = −0.16, *p* = 0.00035). **(D)** *CETP*_rs708272 in subcutaneous adipose tissue (*β* = −0.17, *p* = 0.00063). **(E)** *ABCA1*_rs1883025 in the skeletal muscle (*β* = 0.1, *p* = 0.0012). **(F)** *SIK3*_rs7115583in the skeletal muscle (*β* = 0.07, *p* = 0.05).

Only the gene expressions of some genetic variants selected for hypo-HDL-C were determined in different tissues. The risk allele of *ALDH1A2*_rs588136 and *SIK3*_rs7115583 had a lower expression than that of the non-risk allele in the cortex of the brain and skeletal muscle (Figure 4). However, the risk alleles of *CETP*_rs708272 and *ABCA1*_rs1883025 had a higher expression than the non-risk allele in the arterial appendage of the heart and skeletal muscle (Figure 4).

### The binding affinity of kuwanol E to APOE_rs429358

**Figure 5:**
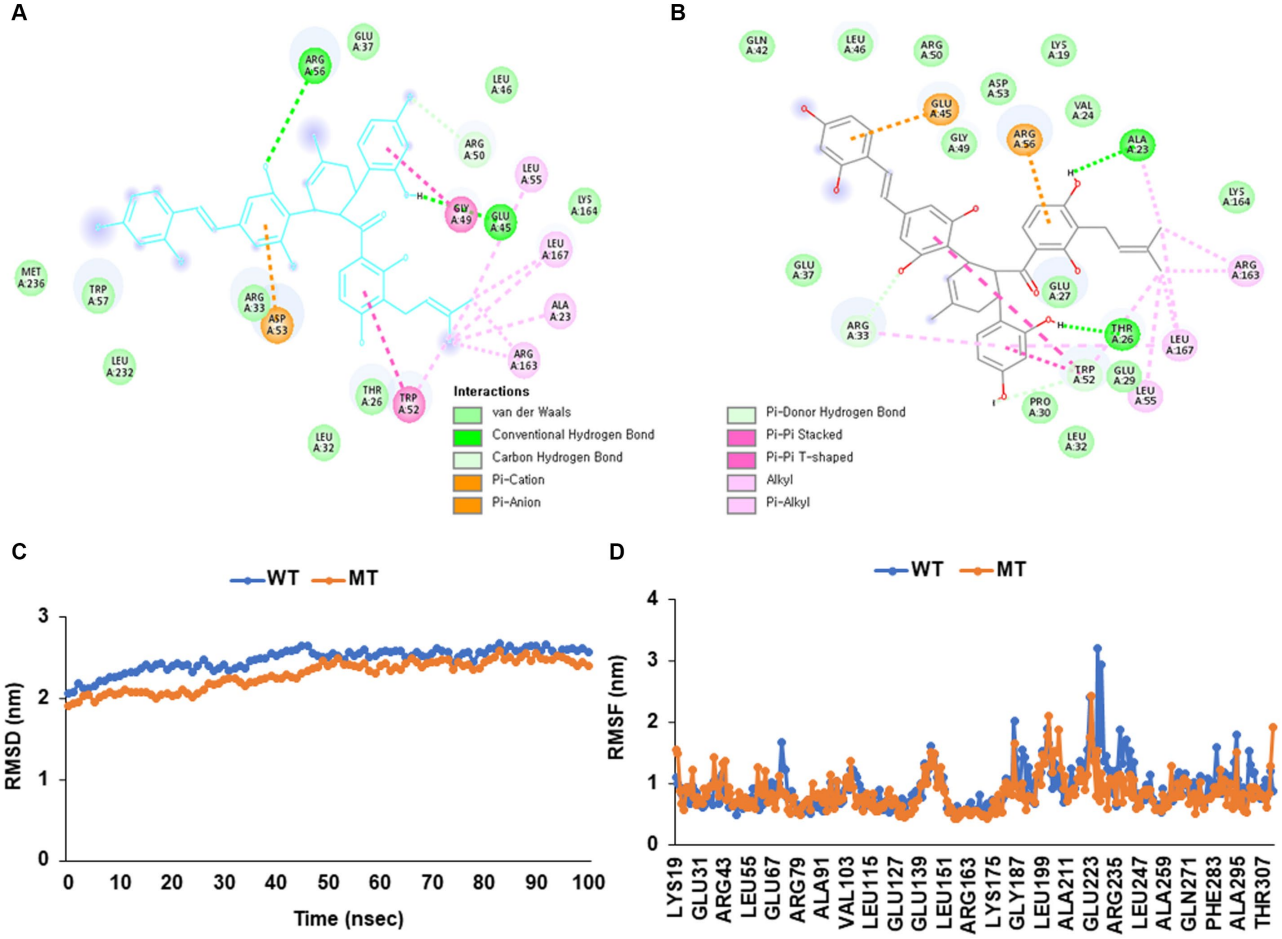
Molecular docking and molecular dynamic simulation (MDS) of Kuwanol E on Apolipoprotein E (*APOE*) wild type (cys130) and mutated type (130arg) in rs429358. **(A)** The interaction force between Kuwanol E and *APOE* _rs429358 wild type. **(B)** The interaction force between Kuwanol E and *APOE* _rs429358 mutated type. **(C)** Root mean square deviation (RMSD) of Kuwanol E on *APOE*_rs429358 wild and mutated types. **(D)** Root mean square fluctuations (RMSF) of Kuwanol E on *APOE*_rs429358 wild and mutated types.

The wild (cys13) and mutated *APOE_*rs429358 (130Arg) exhibited different levels of binding free energy to specific food components (Table 4). Neoacrimarine H, viniferifuran, morellinol, 22-deoxocucurbitacin D, cucurbitacin B, yuccaol A, yuccaol C, pregeijerene, and plantacyanin showed a low binding energy of < −10 kcal/mol to both wild and mutated types of *APOE_*rs429358 (Table 4). However, some food components showed a different binding affinity to wild and mutated types *of APOE_*rs429358. For example, the binding energy of *APOE_*rs429358 with Kuwanol E was −7.3 kcal/mol with the wild type but −10.1 kcal/mol with the mutated type (Table 4). Kuwanol E’s binding to the wild and mutated type of *APOE_*rs429358 is presented in Figures 5A,B.

**Table 4:** Biding energy of food components to *APOE* wild type (WT) and mutated one (MT) in rs429358.

Compounds	Binding energy (kcal/mol)		Herbs
	WT	MT	
epsilon-Viniferin	−10.0	−9.3	Resveratrol dimer
Muzanzagenin	−10.1	−10.1	Wild asparagus (*Asparagus africanus*)
3-Benzoyloxy-6-oxo-12-ursen-28-oic acid	−10.1	−10	*Momordica dioica*
Neoacrimarine H	−10.4	−10.4	*Citrus paradisi* (grapefruit)
Viniferifuran	−10.4	−10.5	Wine grapes (*Vitis vinifera* ‘Kyohou’) and amur grape (*Vitis amurensis*)
22-Deoxocucurbitacin D	−10.4	−10.5	*Lagenaria siceraria* (bottle gourd).
Cucurbitacin B	−10.2	−10.8	Muskmelon
Yuccaol A	−10.6	−10.7	Bark of *Yucca schidigera* (Mojave yucca)
Yuccaol C	−10.4	−10.4	Bark of *Yucca schidigera* (Mojave yucca)
Pregeijerene	−14	−13.9	*Ruta graveolens* (rue) and *Rubus rosifolius* (Mauritius raspberry)
Plantacyanin	−11.1	−11.2	Cucumber
6”-Acetylhyperin 7-rhamnoside	−10	−10.1	Broad bean (*Vicia faba*) leaves
14alpha-Hydroxy-4beta-deoxypaxilline	−10.1	−10.1	*Penicillium paxilli* and *Acremonium lolii*
Cucurbitacide E	−10.4	−10.5	Leaves and fruit of *Cucumis sativus* (cucumber)
Khelmarin D	−10.0	−9.9	*Citrus paradisi* and *Citrus tangerina* (Rutaceae)
WT only			
Delphinidin 3-feruloylglucoside	−10.0	−8.7	Purple tomato
Solacauline	−10	−8.6	*Solanum punae* and *Solanum schreiteri* (Solanaceae)
Isomorellic acid	−10.9	−8.8	*Garcinia morella* (batuan)
MT only			
Quercetin 3-O-xylosyl-glucuronide	−9.0	−10	Green beans
Gambogic acid	−7.9	−10	Gamboge resin (exudate of *Garcinia morella*)
Kuwanol E	−7.3	−10.1	*Morus alba* (white mulberry)

Figures 5C,D show the root mean square deviation (RMSD) and root mean square fluctuation (RMSF) for *APOE_*rs429358 wild and mutated types binding to kuwanol E. RMSD for *APOE_*rs429358 mutated type binding with kuwanol E was sustained close to 3 Å during 100 nanoseconds (Figure 5C). RMSF for *APOE_*rs429358 wild-type binding with kuwanol E also did not exceed 3 nm in the RMSF graph (Figure 5D). These results suggest that kuwanol E was more stably bound to the *APOE_* rs429358 mutated type than the wild type.

### Metabolic functions of the genetic variants associated with hypo-HDL-C

The genetic variants associated with hypo-HDL-C were involved in reverse cholesterol transport, triglyceride-rich lipoprotein particle remodeling, cholesterol storage, regulation of macrophage-derived foam cell differentiation, triglyceride-rich lipoprotein particle remodeling, protein-containing complex remodeling, cholesterol storage, HDL particle remodeling, and phospholipid homeostasis in the biological process of gene ontology (Table 5).

**Table 5:** Pathways related to genetic variants for serum HDL concentration.

Gene set	*N* of genes	Beta	Beta STD	SE	*P* value	*P* value with Bonferroni
GO BP: GO reverse cholesterol transport	17	1.92	0.058	0.231	4.21e-17	6.52e-13
GO BP: GO triglyceride-rich lipoprotein particle remodeling	13	2.20	0.058	0.269	1.42e-16	2.20e-12
GO BP: GO protein-containing complex remodeling	30	1.37	0.055	0.117	4.49e-16	6.95e-12
GO BP: GO cholesterol storage	17	1.69	0.051	0.211	6.70e-16	1.04e-11
GO BP: GO regulation of macrophage-derived foam cell differentiation	30	1.26	0.050	0.163	8.00e-15	1.24e-10
GO BP: GO very low-density lipoprotein particle remodeling	11	2.14	0.052	0.289	6.40e-14	9.90e-10
GO BP: GO positive regulation of cholesterol storage	7	2.60	0.050	0.361	2.98e-13	4.61e-09
GO BP: GO foam cell differentiation	36	1.05	0.046	0.148	6.63e-13	1.03e-08
GO BP: GO protein-lipid complex subunit organization	50	0.90	0.046	0.127	6.97e-13	1.08e-08
GO BP: GO high-density lipoprotein particle remodeling	17	1.518	0.046	0.226	9.22e-12	1.423e-07
GO BP: GO phospholipid homeostasis	10	1.938	0.045	0.296	2.90e-11	4.493e-07

GO, Gene Ontology; BP, biological process.

### Interaction of genetic variants and lifestyle parameters in hypo-HDL-C

**Figure 6:**
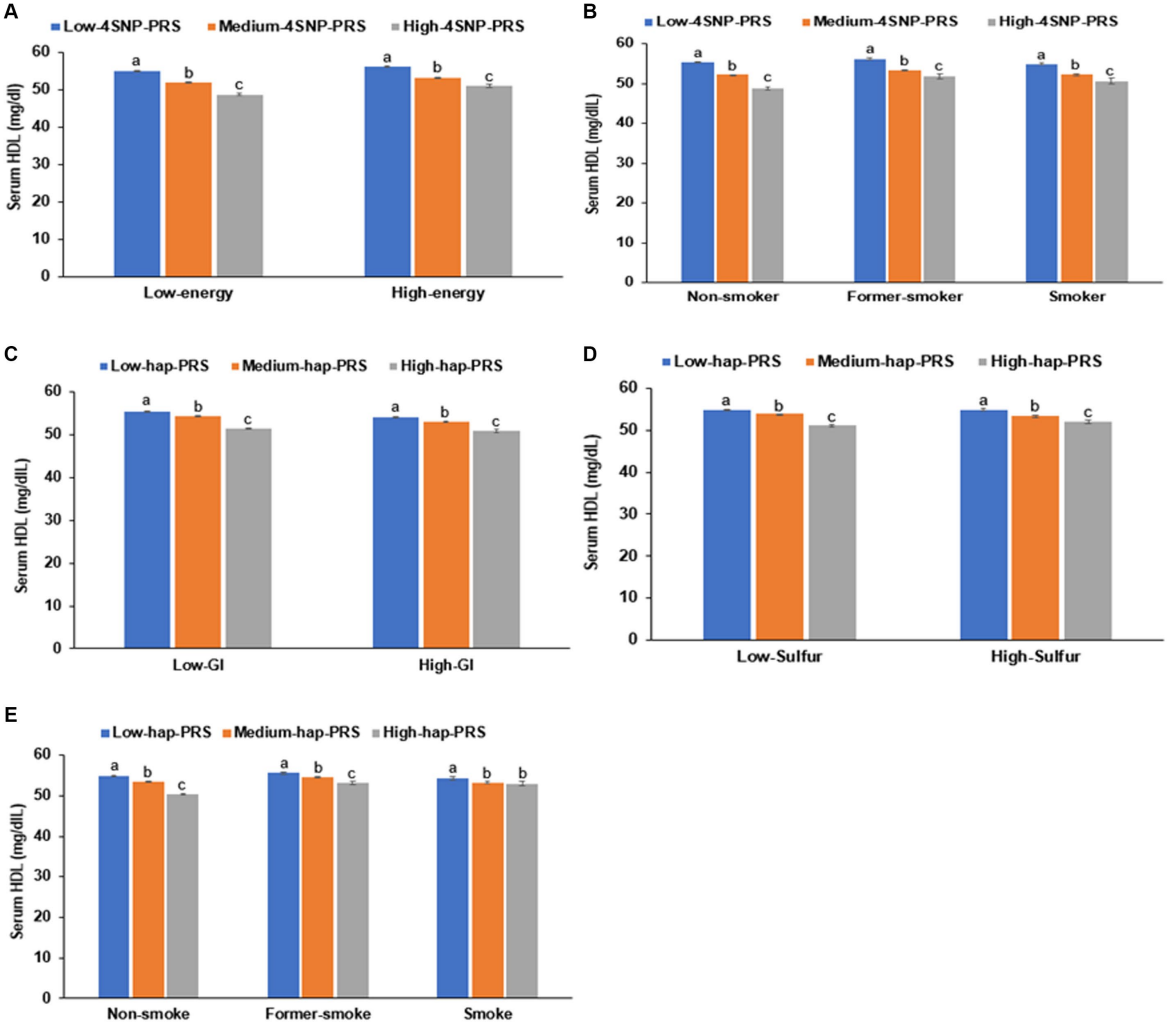
Hypo-HDL cholesterolemia with the polygenic risk score (PRS) according to dietary intake. **(A)** Energy intake in 4-SNP PRS. **(B)** Smoking status in 4-SNP PRS. **(C)** Glycemic index (GI) in haplotype(hap)-PRS of 11q23.3. **(D)** Sulfur microbial diet in hap-PRS of 11q23.3. **(E)** Smoking status in hap-PRS of 11q23.3.

In the interaction between the PRS and lifestyles, the PRS of the 4-SNP model interacted with energy intake (*p* = 0.04) and smoking status (*p* = 0.0006; Table 6). The HDL-C was much lower in the participants in the high-PRS group with a low-energy intake than those with a high-energy intake (Figure 6A). Former and current smokers had lower serum HDL concentration than non-smokers but the PRS effect was smaller in the smokers than non-smokers (Figure 6B). These results indicated that a low-energy intake and non-smoking status did not improve the hypo-HDL-C status in the participants with high-PRS.

**Table 6:** Adjusted odds ratios for the hypo-HDL risk by polygenetic risk scores (PRS) of the best model for gene–gene interaction or haplotype in 11q23.3 after covariate adjustments according to the patterns of lifestyles.

4-SNP PRS	Low- PRS (*n* = 29,317)	Medium-PRS (*n* = 17,592)	High-PRS (*n* = 11,792)	Gene-nutrient interaction *P* value
Low energy^1^ High energy	1	1.566 (1.483–1.653) 1.586 (1.483–1.696)	2.703 (2.372–3.080) 2.143 (1.812–2.533)	0.0426
Low GI^2^ High GI	1	1.552 (1.471–1.637) 1.614 (1.507–1.729)	2.612 (2.292–2.976) 2.267 (1.916–2.682)	0.7382
Low Sulfur^2^ High sulfur	1	1.596 (1.526–1.670) 1.437 (1.278–1.614)	2.521 (2.257–2.815) 2.252 (1.700–2.984)	0.1772
Low DII^2^ High DII	1	1.542 (1.469–1.619) 1.680 (1.544–1.827)	2.436 (2.163–2.745) 2.596 (2.116–3.185)	0.3018
Non-smokers Smokers	1	1.567 (1.499–1.638) 1.674 (1.470–1.906)	2.527 (2.267–2.818) 2.354 (1.724–3.213)	0.0006
Low alcohol^3^ High alcohol	1	1.591 (1.510–1.677) 1.566 (1.458–1.683)	2.576 (2.256–2.941) 2.441 (2.074–2.874)	0.3420

Haplotype	Low-PRS (*n* = 14,156)	Medium-PRS (*n* = 24,671)	High-PRS (*n* = 18,874)	Gene-nutrient interaction *P* value
Low energy^1^ High energy	1	1.169 (1.096–1.248) 1.256 (1.159–1.361)	1.945 (1.749–2.163) 1.813 (1.587–2.072)	0.5180
Low GI^2^ High GI	1	1.218 (1.143–1.299) 1.182 (1.089–1.282)	1.928 (1.735–2.142) 1.855 (1.621–2.123)	0.0025
Low Sulfur^2^ High sulfur	1	1.203 (1.140–1.270) 1.220 (1.059–1.404)	1.974 (1.806–2.159) 1.491 (1.188–1.872)	0.0048
Low DII^2^ High DII	1	1.202 (1.134–1.274) 1.219 (1.102–1.348)	1.949 (1.771–2.144) 1.752 (1.483–2.070)	0.2720
Non-smokers Smokers	1	1.179 (1.118–1.243) 1.436 (1.227–1.680)	1.887 (1.729–2.060) 2.023 (1.563–2.620)	<0.0001
Low alcohol^3^ High alcohol	1	1.138 (1.069–1.211) 1.322 (1.211–1.444)	1.888 (1.699–2.098) 1.978 (1.725–2.268)	0.0953

Values represent adjusted odd ratios and 95% confidence intervals. PRS with 4 SNPs of the best GMDR model or haplotype in 11q23.3 was divided into three categories according to the number of the risk alleles: ≤ 3, 4–5, and ≥ 6 into Low-PRS, Middle-PRS, and High-PRS, respectively. The reference was the low-PRS. Covariates included age, sex, education, income, energy intake, residence areas, daily activity, alcohol intake, smoking status, blood HbA1c, and serum triglyceride concentrations. Cutoff of each variable: 100% for estimated energy requirement (EER)^1^, 33th percentiles^2^, and 20 g/day^3^. GI, glycemic index; DII, dietary inflammation index. ^1^<Estimated energy requirement defined in dietary reference index; ^2^ < 75^th^ percentiles; < 20 g/day; ^3^ < 20 g daily alcohol intake.

The PRS of the haplotype in 11q23.3 interacted with the glycemic index (*p* = 0.003), sulfur microbial diet (*p* = 0.005), and smoking status (*p* < 0.0001) to influence hypo-HDL-C status (Table 6). In participants with a high-GI diet, serum HDL concentration was lower than those with a low-GI diet regardless of haplotype(hap)-PRS but the participants with high-hap-PRS had a much lower serum HDL concentration in Low-GI diet (Figure 6C). In the both low- and high-sulfur microbial diets, participants with a high-hap-PRS had a lower HDL-C than those with a low- hap-PRS, while the PRS impact was greater in the low-sulfur microbial diet than in the high-sulfur microbial diet (Figure 6D). Smokers had lower serum HDL concentrations than non-smokers but the non-smokers with high-hap-PRS showed a remarkably lower than those with low-hap-PRS (Figure 6E). Therefore, low-GI, low-sulfur microbial diet and non-smoking status could not offset the high-hap-PRS impact to decrease serum HDL concentration.

## Discussion

The prevalence of hypo-HDL-C is estimated to be approximately 15–25% in the adult population worldwide (1). Hypo-HDL-C is an independent risk factor for CVD and is associated with an increased risk of coronary artery disease, stroke, and peripheral arterial disease. The risk factors for hypo-HDL-C include age, gender, lifestyle factors (smoking, lack of physical activity, poor diet, and obesity), family history, and certain medical conditions. Age above 55 years, the female gender, higher BMI, waist circumferences, fat mass, hyperglycemia, insulin resistance, hypertension, and hypertriglyceridemia were risk factors for hypo-HDL-C in the present study. Among lifestyle factors, the risk factors were observed to be a high carbohydrate and sodium intake, low protein, vitamin D, coffee, and alcohol intake, low exercise, and smoking. In addition to lifestyle factors, hypo-HDL-C was associated with genetic factors, and the interaction between genetic and lifestyle factors influenced hypo-HDL-C.

In the present study, the PRS of the 4-SNP model and the 11q23.3 haplotype were positively associated with hypo-HDL-C by about 3 times. This was a higher association than the PRS of the 24 SNP model with *p* < 5×10^−8^. These results suggest that the interaction between genetic variants with each other showed a better association with hypo-HDL-C when they were pooled. The selected genetic variants indicated the essential pathways that influence hypo-HDL-C. The SNPs associated with hypo-HDL-C identified in the present study were involved in generating HDL-C, reverse cholesterol transport, triglyceride transport, and cholesterol metabolism in macrophage and foam cells. Pre-β-HDL is formed and secreted from the liver and intestines into the bloodstream. It interacts with ABCA1 to form disc-shaped nascent HDL by the efflux of phosphatidylcholine and cholesterol, and phospholipids from the macrophage and foam cells are transferred into nascent HDL-C through ABCA1 (7). The intracellular cholesterol contents control the expression of the ABCA1 gene. The nascent HDL-C is converted into mature spherical HDL by esterifying free cholesterol by LCAT, reverse cholesterol transport. CETP, mainly released from the liver, is bound to HDL-C in the blood, and it facilitates the movement of cholesteryl ester and triglycerides among HDL, LDL, and very low-density lipoprotein (VLDL). Moreover, increased HDL-C content and activity decreases triglycerides in the serum. Therefore, the expression and mutation of *APOA1, ABCA1, LCAT, CETP*, and *LPL* are involved in HDL-C and triglyceride homeostasis (25). The present study showed that their genetic variants were associated with hypo-HDL-C.

Interestingly, hypo-HDL-C is genetically linked to triglyceride transfer from chylomicron and VLDL. The *APOA1/C3/A4/A5-ZPR1-BUD13* gene cluster is located on chromosome 11q23.3 and modulates LPL activity (26, 27). The cluster is also linked to HDL metabolism. The best model with SNP-SNP interaction included 2 SNPs (*ZPR1*_rs3741297 and *BUD13_*rs180327). The PRS of the 4-SNP model and 11q23.3 haplotype were positively associated with not only hypo-HDL-C but also hypertriglyceridemia. However, the PRS of the 4-SNP model and 11q23.3 haplotype were not associated with other biochemical and anthropometric measurements. This suggests that triglyceride metabolism is closely linked to HDL metabolism. Girona et al. (28) have reported that the triglyceride content in HDL is strongly inversely related to HDL-C and positively associated with the triglyceride contents in chylomicron and VLDL. HDL-C, with high triglyceride content, is small and has a severely abnormal structure (29). Small HDL or HDL with high triglycerides is a marker of cardiovascular disease (29). Therefore, the genetic variants involved in triglyceride metabolism may be linked to the triglyceride movement of not only chylomicron and VLDL but also HDL.

ApoE mediates the binding of lipoproteins, especially VLDL and chylomicron remnants or lipid complexes in the plasma or interstitial fluids, to specific cell-surface receptors (30). In the present study, *APOE_*rs429358, a missense mutation (cys130arg), was positively associated with hypo-HDL-C. Consistent with the present study, *APOE*_rs429358 and rs7412 polymorphisms have been positively associated with hypo-HDL-C, hyper-LDL-C, and hypertriglyceridemia in an earlier study. Furthermore, the lipid profile was linked to cognition in the aging Chinese population (31) and Eastern Europe, as observed in the Health, Alcohol, and Psychosocial Factors in Eastern Europe (HAPIEE) study (32). Therefore, *ApoE*_rs429358 is associated with dyslipidemia, indicating it impacts ApoE activity, thereby modulating the lipid profile.

However, no study has investigated the change in ApoE binding energy with food components according to the rs429358 mutation. Its wild and mutated type proteins showed similar or different binding energies to food components. Among 20,000 food components, 13 food components, mainly polyphenols, had < −10 binding energy with wild and mutated types of *APOE_*rs429358 (cys130arg). Two (solacauline and isomorellic acid) and three food components (quercetin 3-O-xylosyl-glucuronide, gambogic acid, and kuwanol E) had low binding energy with wild-type or mutated-type APOE, respectively. The differences in binding energy between the wild and mutated types were due to conformational changes of the ApoE expressed by the gene with a missense mutation. The changes modified intermolecular binding affinities, such as the conventional hydrogen bonds between food components and the ApoE protein. Kuwanol E known to be present in *Morus alba* had a lowered binding affinity with the mutated type rather than the wild one. Previous studies have shown that *Morus alba* intake protects against dyslipidemia and hepatic liver steatosis in rats and humans (33–35). Lowering the binding energy of ApoE improves its activity by lowering serum HDL-C concentrations.

The relationship between dietary fat and carbohydrate intake and HDL-C is somewhat paradoxical with respect to CVD risk (36). HDL-C decreases by replacing dietary saturated fat with polyunsaturated and monounsaturated fat (36). Furthermore, the switch from dietary fat to dietary carbohydrate reduces HDL-C. The present study showed that high carbohydrate and low-fat intake, regardless of the type of fat, decreased HDL-C (*p* < 0.001). Although fiber intake was lower in the Low-HDL group than in the Normal-HDL group (*p* < 0.05), the dietary glycemic index and sulfur diet index did not differ between the Low-HDL and Normal-HDL groups. Vitamin D intake is a well-known regulator of calcium homeostasis, blood pressure, and glycemia (37). Vitamin D significantly enhances HDL-C to reduce atherosclerotic cardiovascular disease risk scores (38, 39). Consistent with the previous studies (40), vitamin D and calcium intakes were higher in the Normal-HDL group than in the Low-HDL group.

Previous studies have consistently reported that individuals with higher sulfur microbial diet scores related to consuming a high intake of processed meats, liquor, low-calorie drinks, beer, sweets, and desserts and a low intake of mixed vegetables and legumes face an elevated risk of early-onset adenomas and colorectal cancer, with the risk being 1.31 and 1.25 times higher, respectively (41, 42). Furthermore, recent research has highlighted the influence of sulfur microbial diet scores on obesity risk and its impact on metabolic processes, based on data from the UK Biobank (20). However, our current study yielded some intriguing findings. We observed no significant differences in sulfur microbial diet scores between individuals categorized as having Low-HDL and Normal-HDL levels. This discrepancy can be attributed to the distinctive dietary habits of Koreans, where the average sulfur microbial diet scores were notably lower (approximately −35 for men and − 55 for women) compared to Europeans (approximately −0.5). This variation arises from the fact that Koreans consume considerably fewer processed meats and consume more legumes and vegetables than their European counterparts (20). Additionally, our study uncovered a noteworthy interaction between sulfur microbial diet scores and PRS to affect serum HDL concentration. In cases where individuals adhered to a low sulfur microbial diet, those with medium-hap-PRS exhibited a reduced genetic influence on their serum HDL levels. The impact of haplotype genetics was less pronounced in a low-sulfur microbial diet, underscoring the intricate interplay between diet and genetics.

Some studies have demonstrated that genetic variants related to hypo-HDL-C and lifestyle factors exhibit an interaction to modulate HDL-C. For example, total fat intake interacted with the LPL_rs13702 polymorphism to impact HDL-C (interaction *p* = 0.041). The individuals with the risk allele (G) of *LPL_*rs13702 have significantly higher HDL-C when consuming a high-fat diet (>92 g/day) than those on a low-fat diet (*p* = 0.033) (43). The risk allele of the haplotype in the 12q23 has a positive association with hypo-HDL-C by 1.65 times compared to its non-risk alleles. The risk allele of the haplotype interacts with protein, saturated fat, and polyunsaturated fatty acid intake (16). However, the PRS with 4-SNP and the haplotype 11q23.3 had no interaction with protein and fat intake. On the other hand, there was an interaction of the PRS with 4-SNP and haplotype 11q23.3 with energy intake and microbial sulfur diet, respectively. The PRS of 4-SNP and haplotype did not interact with protein and fat intake but with the sulfur microbial diet containing high in meats, mainly processed meats, and low in vegetables. Therefore, the genetic variants are likely associated with the intake of saturated fat and proteins.

The present study is novel as it has shown that HDL-C was associated with not only cholesterol but also triglyceride transfer from triglyceride-rich lipoproteins. Furthermore, HDL-C was related to the regulation of macrophage differentiation derived from foam cells. The PRS of the 4-SNP and haplotype 11q23.3 interacted with energy and the sulfur-microbial diet score, respectively, to influence low HDL-C. The limitations of the study were as follows: First, the data originated from a cross-sectional study, and although it was well-designed, and the data collection and measurement were well-controlled, the results could not represent cause and effect. Second, HDL subclasses, including HDL particle size, composition, and functionality, were not measured to understand the genetic impact of HDL metabolism and its interaction with lifestyles. Third, daily food intake was estimated from the SQFFQ, including 106 common Korean foods and dishes. The SQFFQ included some bias for the usual food intake, although it was checked with a three-day record four times. Third, genetic variants were estimated with a customized K-chip for Koreans (Axiom Biobank plus Genotyping Array, KNIHv1.1) (44). The genetic variants might not include some genetic variants related to metabolic diseases.

In conclusion, adults with hypo-HDL-C had a 1.4-fold higher risk of CVD. Those with a high PRS of *ZPR1*_rs3741297, *CETP*_rs708272, *BUD13*_rs180327, and *ALDH1A2*_rs588136 or the haplotype 11q23.3 were positively associated with the risk of hypo-HDL-C by about 3 times. The PRS of the 4-SNPs and haplotypes interacted with energy intake and sulfur-microbial scores, affecting hypo-HDL. The wild type of *APOE*_ rs429358 (cys130) lowered the binding energy to polyphenols somewhat differently than the mutated ones (130arg). Therefore, adults with a genetic risk for hypo-HDL need to modulate their diet to reduce their risk. Our study demonstrates the clinical relevance of genetic variants associated with hypo-HDL-C in a large cohort of middle-aged Asian adults. These findings highlight the diagnostic value of incorporating genetic risk assessment into managing hypo-HDL-C and, eventually, cardiovascular disease. Individuals identified with a genetic predisposition to hypo-HDL-C can benefit from targeted interventions, such as lifestyle modifications such as low sulfur microbial and glycemic diets and non-smoking and early screening, to mitigate their increased risk of myocardial infarction and stroke. Implementing personalized risk assessments based on genetic factors has the potential to enhance preventive strategies and improve patient outcomes in clinical practice.

## Data availability statement

Publicly available datasets were analyzed in this study. This data can be found here: http://www.kbn.re.kr/kbn/main.do.

## Ethics statement

The institutional review boards (IRB) of the Korea National Institute of Health and Hoseo University approved the KoGES and the present study (KBP-2015-055 and 1041231-150811-HR-034-01, respectively). All participants signed written informed consent. The studies were conducted in accordance with the local legislation and institutional requirements. The participants provided their written informed consent to participate in this study.

## Author contributions

SP and M-SK: conceptualization. HH, HY, and MK: methodology. M-SK and DJ: resources. HH and KL: data collection and analysis. SP: writing—original draft preparation. HH and M-SK: writing—review and editing. M-SK and SP: supervision. All authors have read and agreed to the published version of the manuscript.

## Footnotes

^1^https://github.com/Kyoko-wtnb/FUMA-webapp/

^2^https://biit.cs.ut.ee/gprofiler/snpense

^3^http://genemania.org/

^4^https://gtexportal.org/home/testyourown, accessed on July 19, 2022.

^5^https://zhanggroup.org/I-TASSER/

^6^https://proteins.plus/
