# Supplementary material for: Interaction of energy and sulfur microbial diet and smoking status with polygenic variants associated with lipoprotein metabolism
Source: Front Nutr. 2023 Oct 4;10:1244185. doi: 10.3389/fnut.2023.1244185 (PMC10582641; doi:10.3389/fnut.2023.1244185)

Table S1. List of the foods in each dietary pattern using principal component analysis.

	Korean-balanced diet	Plant-based diet	Western-style diet	Rice-main diet
Rice	-3	-7	4	93 *
Grains	9	-4	-2	-93 *
Noodles	2	2	62 *	3
Breads	-7	35	53 *	-5
Cookies	-7	31	30	6
Beans	32	48 *	3	2
Potatoes	24	50 *	5	-3
Kimchi	50 *	1	-1	-3
Egg	8	43 *	15	5
Fast food	-4	15	76 *	-4
Green vegetables	67 *	42 *	-2	-2
White vegetables	70 *	29	2	2
Mushrooms	49 *	35	-6	-4
Fatty fish	53 *	23	11	0
White fish	66 *	17	12	0
Crabs	49 *	4	19	1
Processed meats	18	15	6	-1
Meats	46 *	-7	41 *	8
Soup	16	4	65 *	-5
Chicken	33	-6	39	4
Seaweeds	44 *	41 *	-2	-4
Milk	11	49 *	2	0
Beverages	20	32	6	2
Coffee	10	-1	19	15
Tea	14	-8	24	14
Fruits	19	48 *	-6	-6
Pickles	50 *	-1	4	2
Nuts	-2	50 *	6	-5
Alcohol	18	-28	16	6

Printed values are multiplied by 100 and rounded to the nearest integer. Values greater than 0.4 are flagged by an '*'.

Table S2. Generalized multifactor dimensionality reduction (GMDR) results of multi-locus interaction with genes related to hypo-HDL-cholesterolemia risk

Model 1	TRBA	TEBA	P value	CVC
*ZPR1*_rs3741297	0.5423	0.5423	0.001	10
*CETP*_rs708272 plus model 1	0.5592	0.5578	0.001	9
*BUD13*_rs180327 plus model 2	0.568	0.568	0.001	10
*ALDH1A2*_rs588136 plus model 3	0.5739	0.5733	0.001	10
*LPL*_rs325 plus model 4	0.5792	0.5756	0.001	9
*ABCA1*_rs1883025 plus model 5	0.5851	0.5777	0.001	10
*APOE*_rs429358 plus model 6	0.5932	0.5739	0.001	10
*SIK3*_rs7115583 plus model 7	0.6019	0.571	0.001	9
*CD36*_rs146148222 plus model 8	0.6123	0.5671	0.001	10
*APOA1*_rs12718465 plus model 9	0.6224	0.568	0.001	10

Adjusted for SEX, AGE, BMI, EDU, INCOME, ALCOHOL, SMOKE, EXER, AREA, and energy intake

Table S3. Adjusted means and odds ratio according to PRS of the 4-SNP model selected by GMDR

	Low- PRS (n=29,317)	Medium-PRS (n=17,592)	High-PRS (n=11,792)	Adjusted ORs and 95% CI
HDL (mg/dL)	55.7±0.07[a]	52.1±0.08[b]	48.3±0.27[c***]	2.232 (2.035-2.448)
BMI (kg/m2)	23.9±0.02	23.9±0.02	23.8±0.06	0.939 (0.852-1.034)
Waist (cm)	80.7±0.05	80.7±0.05	80.6±0.17	0.930 (0.831-1.041)
SMI (mg/m)	7.01±0.003	7±0.004	7±0.013	0.899 (0.800-1.011)
Fat mass (%)	28.4±0.02	28.4±0.02	28.3±0.08	0.908 (0.825-1.000)
Total cholesterol (mg/dL)	198±0.21[a]	197±0.22[b]	194±0.78[c***]	0.914 (0.804-1.040)
LDL (mg/dL)	119±0.19[a]	119±0.2[a]	117±0.72[b*]	0.914 (0.804-1.040)
TG (mg/dL)	119±0.49[c]	130±0.51[b]	147±1.8[a***]	1.629 (1.481-1.792)
Serum glucose (mg/dL)	95±0.12	95.2±0.12	95.8±0.43	1.030 (0.899-1.181)
HbA1c (%)	5.71±0.006	5.71±0.006	5.74±0.021	1.002 (0.856-1.173)
SBP (mmHg)	122±0.08	122±0.09	123±0.3	0.994 (0.901-1.097)
DBP (mmHg)	75.7±0.06	75.7±0.06	76±0.2	1.014 (0.868-1.185)
GFR (mL/min/1.73 m^2)	85.9±0.09	86±0.09	85.1±0.34	1.062 (0.834-1.353)
ALT (U/L)	22.3±0.13	22.5±0.14	22.7±0.49	1.165 (1.013-1.341)
AST (U/L)	23.7±0.14	23.9±0.14	23.9±0.51	1.037 (0.832-1.291)

Values represent adjusted means and standard errors after adjusting covariates.

[*] Significant differences by gender at P<0.05, [**] at P<0.01, [***] P<0.001.

[a,b,c] Different superscript letters indicated significant differences a1ong the groups in Tukey's test at p<0.05.

Table S4. Adjusted means and odds ratio according to PRS of haplotype in 11q23.3

	Low-PRS (n=14,156)	Medium-PRS (n=24,671)	High-PRS (n=18,874)	Adjusted ORs and 95% CI
HDL (mg/dL)	55.7±0.07[a]	53.6±0.06[b]	48.9±0.19[c***]	2.232 (2.035-2.448)
BMI (kg/m2)	23.9±0.02	23.9±0.01	23.9±0.04	0.939 (0.852-1.034)
Waist (cm)	80.8±0.07	80.7±0.04	80.7±0.12	0.930 (0.831-1.041)
SMI (kg/m)	6.47±0.006	6.46±0.004	6.46±0.011	0.899 (0.800-1.011)
Fat mass (%)	28.4±0.02	28.4±0.02	28.3±0.08	0.908 (0.825-1.000)
Total cholesterol (mg/dL)	198±0.21[a]	197±0.22[b]	194±0.78[c***]	0.914 (0.804-1.040)
LDL (mg/dL)	119±0.28[a]	119±0.17[a]	116±0.50[b*]	0.914 (0.804-1.040)
TG (mg/dL)	107±0.69[a]	126±0.41[b]	170±1.24[c***]	1.629 (1.481-1.792)
Serum glucose (mg/dL)	95±0.17	95.1±0.10	95.7±0.30	1.030 (0.899-1.181)
HbA1c (%)	5.71±0.01	5.71±0.01	5.73±0.02	1.002 (0.856-1.173)
SBP (mmHg)	122±0.12	122±0.07	122±0.21	0.994 (0.901-1.097)
DBP (mmHg)	75.7±0.08	75.7±0.05	75.8±0.14	1.014 (0.868-1.185)
GFR (mL/min/1.73 m^2)	86.1±0.13	85.9±0.08	85.6±0.24	1.062 (0.834-1.353)
ALT (U/L)	22.5±0.19[b]	22.2±0.11[b]	23.6±0.34[a***]	1.165 (1.013-1.341)
AST (U/L)	23.6±0.2[b]	23.7±0.12[b]	25.2±0.36[a***]	1.037 (0.832-1.291)

Values represent adjusted means and standard errors after adjusting covariates.

[*] Significant differences by gender at P<0.05, [**] at P<0.01, [***] P<0.001.

[a,b,c] Different superscript letters indicated significant differences a1ong the groups in Tukey's test at p<0.05.

Figure S1A. Manhattan plot. Figure S1B. Q-Q plot.

S1A

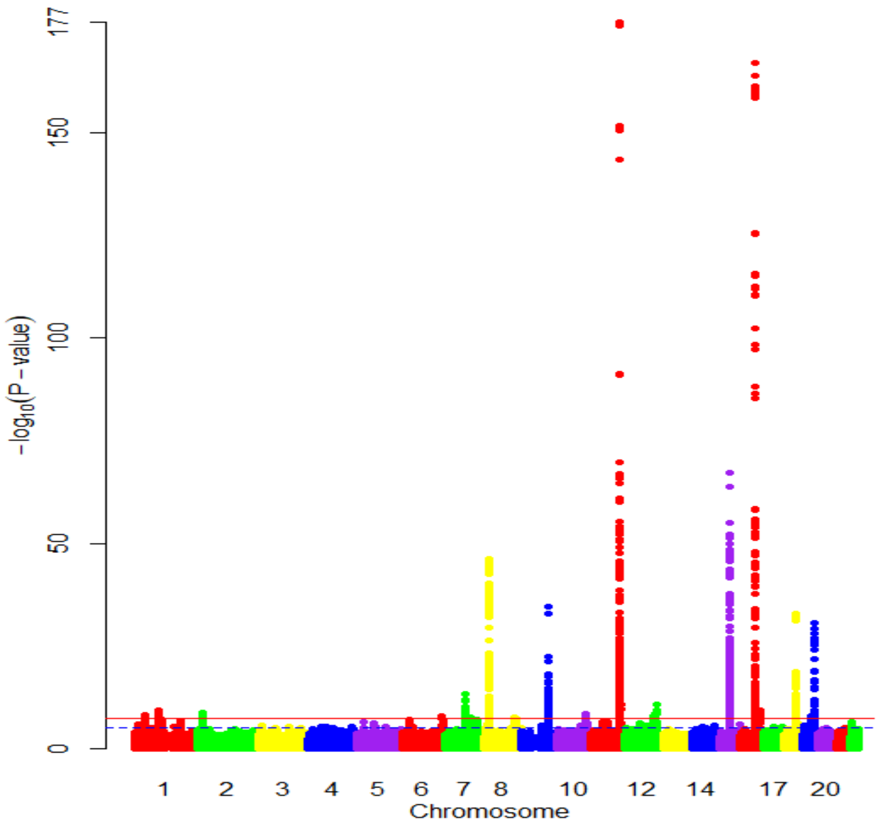

Fig. S1B

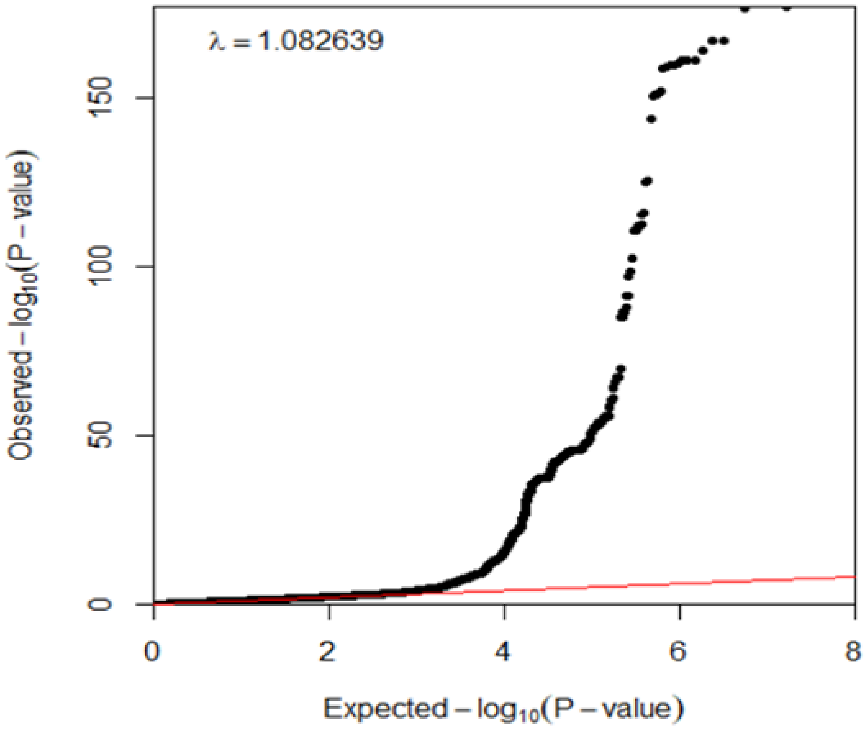

Fig. S2. Linkage disequilibrium in 11q23.3

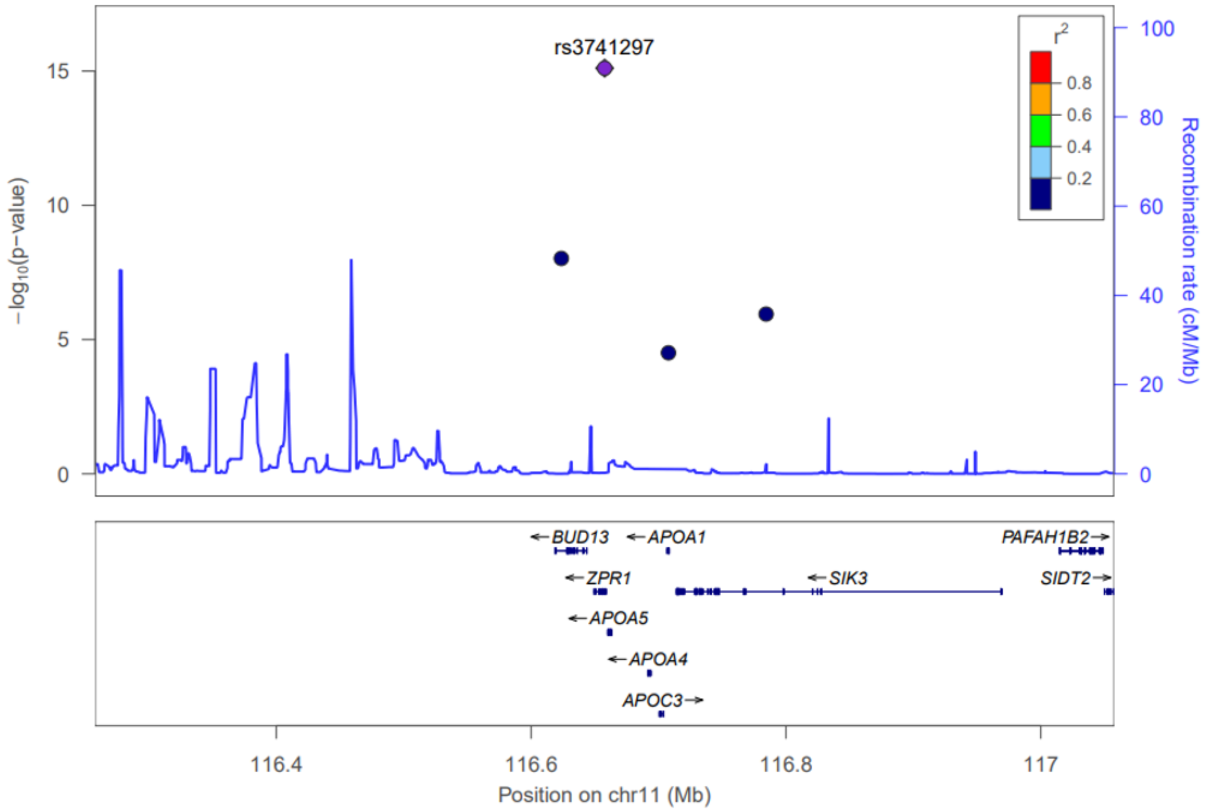

Supplement: Supplementary file 1 [file Data_Sheet_1.PDF]
